# Report of a case of renal hilum Castleman disease complicated with Paget’s disease of the breast

**DOI:** 10.3389/fmed.2026.1836732

**Published:** 2026-06-03

**Authors:** Weidong Yang, Hongmei Li, Dan Shen, Deqiang Gu, Shichao Song, Zhenyu Cui

**Affiliations:** 1 Department of Urology, Affiliated Hospital of Hebei University, Baoding, Hebei, China; 2 Department of Emergency, Affiliated Hospital of Hebei University, Baoding, Hebei, China

**Keywords:** Castleman disease, lymphoproliferative, renal hilum, retroperitoneum, surgery

## Abstract

Renal hilar Castleman disease is a rare lymphoproliferative disorder characterized by non-specific radiological manifestations. It is easily misdiagnosed as primary renal or adrenal malignant tumors in clinical practice, which may consequently lead to unnecessary radical nephrectomy. A 51-year-old female with a history of right breast cancer treated with mastectomy and adjuvant endocrine therapy 7 years prior presented with right nipple erosion. Biopsy confirmed Paget’s disease, and preoperative staging with contrast-enhanced computed tomography (CT) incidentally revealed a solitary soft-tissue mass adjacent to the left renal hilum and abdominal aorta, with a maximum diameter of 8.5 cm × 6.2 cm and moderate heterogeneous enhancement. Strip-like and punctate calcifications were observed within the lesion, accompanied by reactive lymph nodes at the periphery and compression as well as displacement of the left renal vessels. The patient presented no specific clinical symptoms related to the retroperitoneal mass. According to comprehensive preoperative assessment and multidisciplinary consultation, the patient underwent complete radical resection of the renal hilar mass combined with left nephrectomy. Postoperative histopathological examination and immunohistochemical staining confirmed the diagnosis of hyaline-vascular type Castleman disease. The patient recovered uneventfully after surgery, and no local recurrence or distant metastasis was observed during the 12-month follow-up, with a favorable long-term prognosis. This case highlights the radiological difficulties in differentiating renal hilar Castleman disease from malignant renal tumors and emphasizes the necessity of standardized preoperative multimodal evaluation, which helps avoid excessive surgical intervention such as blind radical nephrectomy for unexplained retroperitoneal masses.

## Introduction

Castleman disease (CD) is a group of rare lymphoproliferative disorders characterized by distinctive histopathological features (1). It has an incidence <1/100,000, with a bimodal age distribution (30∼40 years and 60∼80 years) and a similar incidence in all genders (2). Retroperitoneal Castleman disease is uncommon, though an increasing number of cases involving the kidney and perirenal structures have been reported in recent years (3, 4). As the renal hilum is adjacent to vital blood vessels, such as the renal arteries and veins and the abdominal aorta, it is prone to vascular compression after the onset of CD, posing greater challenges for preoperative diagnosis and surgical treatment, and related case reports remain extremely scarce. At the same time, the lack of specific clinical manifestations and imaging characteristics makes preoperative diagnosis particularly difficult, often leading to confusion with common adrenal tumors, such as adrenal cortical carcinoma, pheochromocytoma, neurogenic tumors, and metastatic tumors (5, 6). This report presents a rare case of incidental discovery of left renal hilum CD in a patient with a history of breast cancer and newly diagnosed Paget’s disease, in which an analysis of its clinical, imaging, and pathological features were conducted in conjunction with a literature review, and diagnosis/treatment strategies and surgical key points were discussed, aiming to provide references for the early diagnosis and precise treatment of such diseases in clinical practice.

## Case report

Patient: A 51-year-old female presented with the complaint of “a mass in the left renal hilum for more than a month.” The patient was postmenopausal, with age at menopause of 50 years. She had no family history of malignant tumors, including breast cancer, ovarian cancer, or other hereditary tumor syndromes. One month prior to admission, the patient had a history of right breast cancer surgery for over 7 years and had experienced right nipple erosion for more than 3 months. As part of the routine preoperative systemic workup for breast and nipple surgery, enhanced abdominal and renal CT was performed to evaluate the general condition and exclude occult intra-abdominal lesions. A renal enhanced CT scan (including three-dimensional reconstruction) showed a mass adjacent to the left renal hilum and the abdominal aorta, accompanied by multiple small lymph nodes, suggestive of a pheochromocytoma or a lymphoid tumor. The patient experienced no abdominal pain or distension; no fever or chills; no nausea or vomiting; no frequency or urgency of urination, or dysuria; no gross hematuria. The patient has a history of hypertension for 7 years, with a maximum blood pressure of 180/100 mmHg. She regularly takes 25 mg of captopril once daily and self-reports adequate blood pressure control. She underwent a cesarean section in 2005 and had a subcutaneous mastectomy with preservation of the nipple-areolar complex for right breast cancer in 2019, with satisfactory postoperative recovery. Following the 2019 surgery, she received adjuvant endocrine therapy with letrozole for 5 years and did not undergo radiotherapy. In 2023, she underwent an enlarged excision of the right nipple-areolar complex due to Paget’s disease, with satisfactory postoperative recovery. No additional adjuvant therapy was administered after this procedure Physical Examination: The patient was alert and in normal spirits; body temperature: 36.2°C; pulse: 81 beats/min; respiration: 20 breaths/min; blood pressure: 148/106 mmHg. No facial acne, moon facies, or buffalo hump, nor excessive hair growth or skin thinning was observed. There was no swelling in the renal areas, and no tenderness or percussion pain was noted in the renal and bilateral ureteral regions.

Renal enhanced CT showed: A soft tissue density mass adjacent to the left renal hilum and the abdominal aorta, measuring 8.5 cm × 6.2 cm at its largest dimension, with moderate heterogeneous enhancement on contrast scans. Additionally, strip-like and punctate high-density calcifications were observed within the mass, multiple lymph nodes were noted at the mass’s margin, and the left renal vessels were compressed, suggestive of a pheochromocytoma or a lymphoid tumor (Figure 1). Renal artery CTA indicated: A mass in the left renal hilum supplied by the left renal artery and the abdominal aorta, with the left renal artery being compressed and displaced. Upon admission, relevant follow-up examinations and laboratory tests were performed, and cardiac/pulmonary examinations suggested no significant abnormalities. The 24-h urinary catecholamines revealed: Epinephrine: 12.11 μg/24 h; norepinephrine: 16.4 μg/24 h; dopamine: 10.06 μg/24 h; plasma renin activity: 1.86 ng/mL; angiotensin I: 3.64 ng/mL (37°C water bath) and 1.78 ng/mL (0°C water bath); angiotensin II: 79.45 pg/mL; adrenocorticotropic hormone (ACTH): <0.5 pg/mL (normal range: <20 pg/mL); blood cortisol: 86.9 ng/mL (normal range: 72.6∼322.8 ng/mL); and aldosterone: 29.66 ng/dL (normal range: 7∼30 ng/dl). Results of routine preoperative tests, including hematology, biochemistry, tumor markers (e.g., CA 15-3, CEA, CA 19-9), and coagulation function, were all within normal ranges. Preoperative Diagnosis: (1) Mass in the left renal hilum; (2) Postoperative Paget’s disease of the right breast; (3) Postoperative ductal carcinoma *in situ* of the right breast. Upon multidisciplinary consultation, given that the mass in the left renal hilum was associated with compression of surrounding tissues and organs, and considering the strong desire of the patient’s family for surgery, surgical treatment was recommended. However, there are significant surgical risks due to the unclear pathological mechanism of the mass and its tight adhesion to surrounding structures. Therefore, open surgery was advised.

**FIGURE 1:**
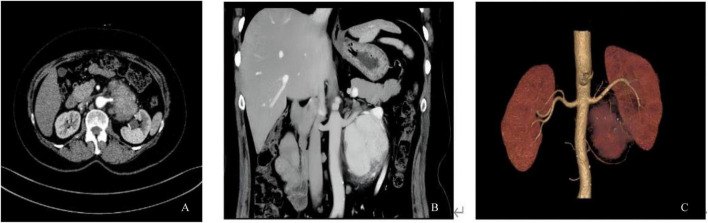
Renal Enhanced CT and Renal Artery CTA. **(A–C)** A soft tissue density mass adjacent to the left renal hilum and the abdominal aorta, measuring 8.5 cm × 6.2 cm at its largest dimension, with moderate heterogeneous enhancement on contrast scans. Additionally, strip-like and punctate high-density calcifications are visible within the mass, and multiple lymph nodes are noted at the mass’s margin. The left renal vessels are compressed by the abdominal aorta and inferior vena cava, and the left renal vessels and the left kidney are compressed and displaced.

Upon completion of preoperative preparations, the surgery was performed under general. Anesthesia on April 27, 2023. Specifically, the abdominal wall was opened layer by layer, revealing a retroperitoneal mass near the abdominal aorta measuring approximately 8 cm × 7 cm. The mass was firm, highly vascularized, and tightly adherent to surrounding vascular structures. The upper pole of the mass encased the renal artery and renal vein, making dissection difficult due to tight adhesion. At the same time, since the mass benefited from a rich blood supply and was close to the abdominal aorta and inferior vena cava, which resulted in significant bleeding during dissection, the nourishing blood vessels of the tumor were ligated with a No. 7 suture before transection. To achieve complete excision of the mass, the left renal artery and vein were transected along the mass’s margin, and the ureter was clamped with a hem-lock, allowing for the complete removal of the mass along with the left kidney. Intraoperative blood loss was approximately 1,100 mL, and 3 units of red blood cell suspension were transfused. The patient’s vital signs remained stable postoperatively. No intraoperative complications such as adjacent organ injury or major vascular laceration occurred. Postoperatively, the patient recovered uneventfully without wound infection, secondary hemorrhage, deep vein thrombosis, or acute renal insufficiency. Serum creatinine remained within the normal range throughout the postoperative period. The drainage tube was removed on postoperative day 3, and the patient was discharged on postoperative day 8. Postoperative pathology (Figure 2) revealed a lymphoproliferative lesion with characteristic histological features of Castleman disease, hyaline-vascular type. Microscopically, the lesion displayed an “onion-skin” arrangement of small lymphocytes surrounding atretic germinal centers, with prominent hyalinized penetrating vessels. Immunohistochemical staining further supported the diagnosis: CD3 and CD5 highlighted a background population of small T cells; CD20 confirmed the presence of B-cell follicles; CD21 delineated expanded follicular dendritic cell networks within the abnormal germinal centers; CD34 demonstrated the prominent vascularity typical of the hyaline-vascular subtype; Ki-67 showed a low proliferation index of approximately 10%, largely confined to the follicular areas; Bcl-2 was negative in the germinal centers, helping to exclude follicular lymphoma; CD38 showed only partial positivity, and Cyclin D1 was negative, ruling out mantle cell lymphoma. Taken together, the histological pattern and immunohistochemical profile were consistent with a diagnosis of Castleman disease, hyaline-vascular type. Despite no significant lesions in the renal parenchyma (the left kidney) and ureter, dilation of the renal pelvis and ureter was noted. Upon follow-up for 12 months, no recurrence was observed in the breast tumor or the tumor in the left renal hilum.

**FIGURE 2:**
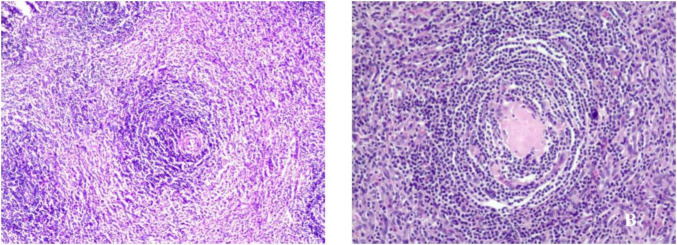
Pathology of Renal Hilum Mass (HE Staining, ×100). Histopathological examination of the renal hilar mass [hematoxylin-eosin staining, **(A)** × 100 **(B)** × 200]. The section shows characteristic “onion-skin” arrangement of small lymphocytes surrounding atretic germinal centers with prominent hyalinized penetrating vessels, consistent with the hyaline-vascular type of Castleman disease.

## Discussion

Castleman disease (CD) consists of several subtypes of lymphoproliferative disorders, and each subtype shows distinct histological features in lymph nodes. On the other hand, various clinical findings and etiological factors make it hard to understand this disease. It was first reported by Benjamin Castleman et al. (7). Pathologically, CD can be further classified into the hyaline vascular subtype of CD (HV-CD), plasma cell subtype of CD (PC-CD), and mixed type of CD. Clinically, CD is divided into unicentric Castleman disease (UCD) and multicentric Castleman disease (MCD). UCD involves a single lymph node region, with most patients exhibiting no systemic symptoms except for lymphadenopathy. By contrast, MCD involves multiple (≥2) lymph node regions (the short diameter of affected lymph nodes ≥ 1 cm) (8). Among all diagnosed cases of CD, 90% are considered unicentric, and the most common subtype is HV-CD, with a higher incidence in females. Retroperitoneal UCD is relatively rare and typically manifests as a solitary mass in the lymphatic drainage pathway, commonly near the kidneys, particularly in the renal hilum (9, 10). CT findings for UCD typically show a well-defined soft tissue mass with homogeneous density, evident enhancement in the arterial phase, and possible punctate calcifications. Hemorrhage and necrosis may occur, which is associated with the tumor’s rich blood supply, good collateral circulation, and the lymphoid tissue’s resistance to necrosis (11). The clinical manifestations of the patient in this case are consistent with UCD. As the most common pathological type of MCD, the plasma cell subtype of CD (PC-CD) is more aggressive and often associated with symptoms such as fever, fatigue, weight loss, anorexia, edema, and multi-regional lymphadenopathy and hepatosplenomegaly. CT findings typically reveal multiple, similarly sized, oval to round low- to intermediate-density masses, most of which are uniformly enhanced; and larger lesions may show irregular enhancement (12). Given this, the definitive diagnosis of Castleman disease relies on pathological examination and immunohistochemistry (13). It should be emphasized, however, that none of the aforementioned imaging features are specific to Castleman disease, as they overlap considerably with those of primary renal and adrenal malignancies. This diagnostic ambiguity may lead to radical nephrectomy being performed for what ultimately proves to be a benign lymphoproliferative lesion—an outcome that carries particular consequences for patients with preexisting renal impairment or hypertension, in whom the unnecessary loss of functional nephrons may accelerate renal functional decline. In the present case, despite comprehensive laboratory exclusion of pheochromocytoma and adrenal cortical carcinoma, definitive diagnosis was still only achieved postoperatively. For similar cases in which CD is suspected preoperatively, core needle biopsy may be considered, as a tissue diagnosis could potentially permit nephron-sparing surgery and avert avoidable nephrectomy.

Although retroperitoneal Castleman disease is uncommon, primary renal or perirenal involvement has been documented with increasing frequency in the literature. A targeted review of reported cases reveals several recurrent clinical and radiological patterns that are instructive for differential diagnosis. Li et al. described a case of Castleman disease arising from the renal parenchyma that was initially misdiagnosed as renal cell carcinoma, emphasizing that the presence of characteristic “onion-skin” lymphoid architecture and hyalinized vessels on core biopsy can facilitate a preoperative diagnosis and avert unnecessary radical nephrectomy (14). Similarly, another case series highlighted that perirenal Castleman disease frequently demonstrates intense arterial-phase enhancement on contrast-enhanced CT, with punctate or arborizing calcifications that may mimic adrenal or renal neoplasms, yet the absence of vascular invasion and the presence of a feeding vessel from the renal artery may serve as distinguishing features (15, 16). In a report by Radfar et al., a renal hilar Castleman tumor with a radiographic appearance nearly identical to that of our case was successfully managed by tumor enucleation with preservation of the renal unit after intraoperative frozen section excluded malignancy, underscoring the importance of intraoperative pathological consultation when preoperative biopsy is not feasible or conclusive (17). Of particular relevance, Nolan et al. reported a case with imaging findings highly consistent with ours—a hypervascular mass adjacent to the renal hilum with vascular encasement—further confirming that such vascular involvement, while surgically challenging, does not necessarily indicate malignant behavior (18). These reports reinforce the notion that Castleman disease should be included high in the differential diagnosis for any well-circumscribed, hyperenhancing retroperitoneal mass in proximity to the kidney, especially when laboratory workup for endocrine tumors is negative. Furthermore, a growing body of evidence suggests that nephron-sparing resection, rather than routine radical nephrectomy, may be appropriate for these benign lesions when technically feasible, highlighting the critical need for accurate preoperative or intraoperative diagnosis. In terms of treatment, as UCD is fully encapsulated and rarely invaded by surrounding tissues, it generally boasts a good prognosis following radical excision, and radiation therapy may be considered if complete resection is not feasible (19, 20). By contrast, MCD demonstrates a poor prognosis, with a 5-year mortality rate of 23%–49%, and the current treatment strategies for it primarily involve combined therapies including targeted therapy, immunotherapy, chemotherapy, and radiation therapy. Single symptomatic treatment is also recommended, along with the use of targeted IL-6 therapies (e.g., Siltuximab or Filgotinib) for patients with specific indications (21, 22). An intriguing aspect of this case is the coexistence of breast cancer, Paget’s disease, and renal hilar Castleman disease in a single patient, raising the question of whether shared mechanisms contributed to their concurrent development. Although a definitive causal link cannot be established from a single case, several potential connections merit consideration. First, chronic inflammation and immune dysregulation may represent a common pathway. Interleukin-6 (IL-6), a key cytokine in Castleman disease pathogenesis, is also implicated in breast cancer progression and endocrine resistance, providing a plausible biological link between the lymphoproliferative and neoplastic conditions. Second, the patient’s postmenopausal status and long-term letrozole therapy profoundly altered her hormonal milieu; whether such changes influence the development of lymphoproliferative disorders warrants further investigation. Third, Paget’s disease of the nipple shares a common neoplastic origin with the underlying ductal carcinoma *in situ*, as observed in this case. While the concurrence of these three conditions may be coincidental, the rarity of this combination merits documentation, and further case accumulation may help elucidate potential biological associations.

Castleman disease (CD) *per se* is a rare condition, and the occurrence of Paget’s disease following breast cancer surgery combined with CD of the renal hilum is even more uncommon. The preoperative diagnosis of this disease is challenging, which can be easily misdiagnosed as a pheochromocytoma or a lymphoid tumor in the renal hilum based on clinical manifestations, laboratory results, and imaging characteristics. In this case, pheochromocytoma was excluded due to the absence of significant elevations in 24-h urinary catecholamines and the lack of typical symptoms such as paroxysmal hypertension. Additionally, since cortisol, aldosterone, and adrenocorticotropic hormone levels were all within normal ranges, along with no manifestations of Cushing’s syndrome observed, adrenal cortical carcinoma was excluded, leading to a pathological diagnosis of CD. On the other hand, surgical resection is considered the preferred therapy for unicentric Castleman disease (UCD) and typically exhibits a good prognosis. However, tumors are often large in diameter, particularly those located in the renal hilum, which can compress the renal vessels and abdominal aorta, thereby increasing the difficulty of laparoscopic surgery. In this case, the tumor was large, highly vascularized, tightly adherent to the abdominal aorta and inferior vena cava, and encircled the renal artery and vein, resulting in significant bleeding during dissection. Therefore, it is necessary to carry out a careful preoperative assessment of surgical difficulty, and the selection of an appropriate surgical approach can reduce intraoperative bleeding. In this case, the preoperative renal enhanced CT with three-dimensional reconstruction clarified the anatomical relationship between the tumor and the abdominal aorta and renal vessels, ultimately providing precise guidance for open surgery and ligation of the tumor’s nourishing blood vessels.

## Conclusion

For soft tissue masses in the renal hilum of unknown origin, especially in patients with a history of malignant tumors, clinicians should include CD in the differential diagnosis, along with the combination of imaging, laboratory tests, and pathological biopsy, aiming to provide a definitive diagnosis. In this regard, adequate preoperative assessment of the tumor’s anatomical relationship with surrounding vessels and the selection of appropriate surgical techniques can improve surgical success rates and enhance patient outcomes.

## Data Availability

The raw data supporting the conclusions of this article will be made available by the authors, without undue reservation.
